# SiO_2_‑Amine Materials from Geothermal Silica Waste with Potential Application in Direct CO_2_ Air Capture

**DOI:** 10.1021/acsomega.6c05141

**Published:** 2026-09-30

**Authors:** Flavia Gutiérrez-Muiña, Rosa-María Ramírez-Zamora, Brenda Alcántar-Vázquez

**Affiliations:** Instituto de Ingeniería, Universidad Nacional Autónoma de México, Avenida Universidad 3000, Coyoacán C.P., 04510 CDMX, Mexico

## Abstract

Direct CO_2_ air capture (DAC) has become a key strategy for tackling climate change, as it can help reduce the concentration of this greenhouse gas (GHG) in the atmosphere, which, as of July 2026, has risen to 429.12 ppm. Most DAC techniques are based on adsorption processes, in which sorbents must have high adsorption capacity, good selectivity, easy regeneration, low energy penalty, suitable adsorption–desorption kinetics, excellent chemical stability, and low cost. Amine functionalization of porous materials has been proposed to improve CO_2_ adsorption capacity and selectivity, and using waste to obtain economically viable adsorbents has attracted interest. Thus, this research aims to develop an efficient SiO_2_-amine adsorbent for direct CO_2_ capture from air using geothermal silica waste. The silica waste comes from two Mexican geothermal power plants. Silica waste was characterized by XRF, XRD, and N_2_ adsorption–desorption, then functionalized with TEPA, PEHA, and PEI by wet impregnation. The resulting SiO_2_-amine materials were characterized by FT-IR and TG. In addition, CO_2_ capture tests were carried out at 25, 30, and 35 °C with 400 ppm of CO_2_. CO_2_ uptake ranged from 0.17 to 0.65 mmol CO_2_/g, with the CT-TEPA material achieving the highest value at 30 °C (0.65 mmol CO_2_/g). The kinetic analysis showed that the nonlinear PSO model adequately described the temporal evolution of CO_2_ capture. Stability evaluation over 10 consecutive adsorption–desorption cycles shows a 3.09% mass loss, mainly due to amine decomposition during desorption. This study systematically evaluates the influence of waste origin, surface treatment, and amine type on adsorbent performance under representative DAC conditions, offering an alternative strategy for developing CO_2_ capture materials from locally available waste resources.

## Graphical abstract

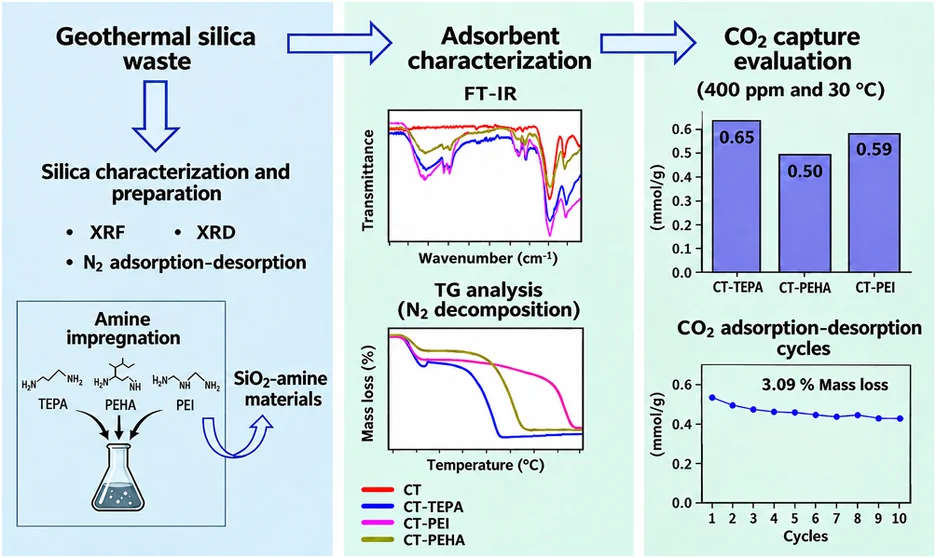

## 1 Introduction

Industrial and technological development since the second half of the 20th century has led to global changes that directly affect the climate. Among these changes, the increase in GHG concentrations, mainly CO_2_, from burning fossil fuels (coal, oil, and natural gas), is a major concern because it raises Earth’s average temperature, alters weather patterns, and affects various ecosystems and human life. CO_2_ is the most dominant GHG in Earth’s current atmosphere: its emissions account for 80% of total GHG emissions, and its concentration has been increasing (429.12 ppm in July 2026).^1^ The sectors with the highest contributions are energy (26%), followed by transport (15%), industry (11%), and agriculture (11%).^2^ Therefore, it is necessary to look for solutions to reduce the concentration of this gas in the atmosphere.

The International Energy Agency proposes three main alternatives to reduce CO_2_: improving energy efficiency, using renewable energy sources and developing CO_2_ capture methods.^3^ The latter is presented as a potential way to rapidly and on a large scale reduce CO_2_ emissions, although the scientific community recommends implementing all strategies together.

CO_2_ capture-separation systems can be divided into four technologies: precombustion, oxycombustion, postcombustion and DAC.^4^ The first three are mainly aimed at collecting the CO_2_ generated at point sources (energy installations, industries) before it is emitted into the atmosphere, while DAC allows the concentration of this gas to be reduced once it has been emitted.^5^ Absorption and adsorption are the CO_2_ capture methods most widely used and among the most advanced in terms of development. In DAC technologies, CO_2_ is removed from the air primarily by adsorption, with the process divided into two sequential stages: adsorption and desorption.

In DAC processes, it is important to identify materials that meet the following criteria: high gas adsorption capacity, good selectivity, easy regeneration, low energy penalty, fast adsorption and desorption kinetics, excellent chemical stability, and low cost.^6^ The main adsorbents used include activated carbon, zeolites, porous polymers, metal–organic frameworks (MOFs), silica-based materials, alkali metals, metal oxides, carbonates and layered double hydroxides (LDH).^7^ To increase their adsorption capacity and selectivity toward CO_2_, some studies propose chemical modifications to the surface of these materials, generally incorporating basic groups (amines) that interact effectively with CO_2_. Among the most commonly used amine species for this purpose are pentaethylenehexamine (PEHA), tetraethylenepentamine (TEPA), polyethylenimine (PEI), triethylenetetramine (TETA), diethylenetriamine (DETA), diethanolamine (DEA) and monoethanolamine (MEA).^8–10^

In 2010, Belmabkhout et al. reported a mesoporous silica grafted with triamine (TRI-PE-MCM-41) that achieved approximately 0.98 mmol CO_2_/g at 400 ppm and 22 °C, about twice that of zeolite 13X under dry conditions. The material also exhibited fast adsorption kinetics, high CO_2_ selectivity and good cyclic stability.^11^ Similarly, in 2012, Choi et al. grafted approximately 5.5% of ethylenediamine (ED) onto the MOF Mg/DOBDC to evaluate its CO_2_ capture performance under ambient conditions. Before grafting, the material exhibited a CO_2_ uptake of 1.35 mmol CO_2_/g in the first cycle, which decreased by about 20% to 1.06 mmol CO_2_/g after four cycles. In contrast, the ED-MOF Mg/DOBDC achieved 1.51 mmol CO_2_/g in the first cycle and showed excellent stability, maintaining capacities of 1.50, 1.54, and 1.55 mmol CO_2_/g over consecutive cycles.^12^ Darunte et al. evaluated polyamine-functionalized MIL-101­(Cr) using tris­(2-aminoethyl)­amine (TREN) and poly­(ethylenimine) (PEI) with loadings ranging from 0.18 to 1.76 mmol_amine_/g_MOF_. They showed that TREN-modified samples achieved high CO_2_ uptake (up to 2.8 mmol CO_2_/g) but were unstable. In contrast, MIL-101­(Cr) + PEI provided a more balanced performance, with a CO_2_ uptake of approximately 1.35 mmol CO_2_/g and good cyclic stability.^13^

Table 1 summarizes the CO_2_ capture capacities reported in studies published since 2019, primarily focused on developing and evaluating chemical adsorbents for DAC.

**Table 1:** CO_2_ Capture Capacity of Amine-Impregnated Solid Adsorbents in DAC Studies^10^

support	amine	amine loading	adsorption conditions (*T*_ads_ (°C)/ ppm of CO_2_)	CO_2_ capture (mmol CO_2_/g)	desorption conditions	stability	refs
SBA-15	DT	0.5–15 SiOH/nm^2^	45/400	0.64			^14^
cellulose acetate fiber and silica	PEI	10 wt %	35/395	2.9	110 °C/2 h	good stability	^15^
SBA-15	PPI	8.17 mmol N/g	35/400	0.31			^16^
SBA-15	PPI	14 mmol N/g	30/400	1.85	110 °C/30 min/He (90 mL/min)	no significant changes (20 cycles)	^17^
Ph-3-SBA-15	ED	16.7 mmol N/g	35/400	1.9	90 °C/10 min/He (90 mL/min)	no significant changes (25 cycles)	^18^
TTE	PEI	30 wt %	20/400 (presence of moisture)	1.14	120 °C/30 min/H_2_O (2 L/min)	no significant changes (25 cycles)	^19^
SBA-15	PEI	75 wt %	25/400	1.90			^20^
MIL-101(Cr)	PEI	50 wt %	25/400	1.81			^21^
MIL-101(Cr)	TEPA	50 wt %	25/400	2.14			
APTES-CeO_2_	APTES	2 wt %	30/400	0.50	250 °C/1 h/He	no significant changes (10 cycles)	^22^

Another criterion to consider when selecting the adsorbent for DAC is that it should be economically viable, so the use of industrial or domestic solid waste has become an alternative for the development of adsorbents, which not only leads to the development of materials at a relatively low cost, but also eliminates waste at the same time.^23^ Various studies show that, to develop adsorbents from waste, it is important to consider raw material selection (availability, properties, and cost), preparation methods and their effects, physicochemical properties and regeneration conditions of the adsorbents, the effects of other gas components, and the environmental impact of the waste materials.^7^ Among the most commonly used waste for this purpose are biomass waste,^24^ fly ash generated by heat combustion,^25,26^ construction and demolition waste,^27^ food waste (rice husks, shrimp shells), metallurgical slags, and, in recent years, geothermal waste due to its high silica content (more than 80%).^28^

Zhang et al. reported that activating bamboo biochar with potassium hydroxide greatly optimizes its morphology and texture, increasing the surface area and resulting in a CO_2_ adsorption capacity of 0.052 mmol/g.^29^ In 2023, Liu et al. evaluated activated carbons derived from agricultural waste, including sugar cane bagasse, corn straw and sawdust, obtaining CO_2_ uptakes of 0.70, 0.75, and 0.71 mmol CO_2_/g, respectively, at a low CO_2_ concentration (≈500 ppm). These results highlight the feasibility of converting lignocellulosic waste into effective, low-cost CO_2_-capture adsorbents.^30^ More recently, Liu et al. investigated municipal wastewater sludge-derived activated carbon impregnated with 20 wt % TEPA, achieving a CO_2_ capture of 1.17 mmol CO_2_/g under ambient conditions, due to enhanced chemisorption via amine-CO_2_ interactions.^31^

Among industrial waste, geothermal plants generate large amounts of waste, including brine and solids deposited in pipes that transport water from the Earth’s crust to the surface.^28^ It has been reported that this type of waste is disposed of annually with no planned application. Although comprehensive global inventories of geothermal solid waste are not currently available, regional studies report substantial waste generation; for instance, geothermal power plants in the Salton Sea Geothermal Resource Area produce approximately 79,800 t of solid waste annually, underscoring the need for appropriate waste management strategies.^32^

Geothermal silica waste mainly comprises amorphous silica (>80%) with low sodium and potassium chloride.^33^ This waste has been investigated for several value-added applications, including its uses as a silica source for alkaline silicate solutions, alkali-activated binders, geopolymers, and Portland cement replacement,^34^ as well as for the synthesis of silica-based materials such as xerogels and functional fillers in composite materials.^35,36^ These studies highlight the potential of geothermal silica waste as a sustainable raw material for advanced material development. Therefore, this work aims to develop an efficient SiO_2_-amine adsorbent material from geothermal silica waste for direct CO_2_ air capture.

## 2 Experimental Section

Silica waste comes from two Mexican geothermal power plants, labeled H and C. Before use, the waste was ground and washed with deionized water to remove impurities.

### 2.1 Citric Acid Treatment of Silica Waste

A citric acid treatment was performed on the washed silica to modify its textural properties. 50 mL of a 5 wt % citric acid solution was added to 5 g of each washed silica sample. The mixture was stirred, heated to 95 °C for 2 h, filtered, and rinsed with deionized water.^37^ The resulting solid was dried at 100 °C for 24 h. The dried materials were then ground and calcined at 700 °C for 30 min.

### 2.2 SiO_2_-Amine Materials Preparation

SiO_2_-amine materials were obtained by wet impregnation of 20 wt % TEPA, PEI or PEHA on washed and acid-treated silica waste. First, the amine was dispersed in 10 mL of methanol with constant stirring for 1 h. Subsequently, the silica sample was added, and the mixture was stirred at room temperature for 4 h. The resulting material was dried at 60 °C in a rotary evaporator (150 rpm) until the methanol was completely removed. Finally, the obtained powder was dried overnight under vacuum at room temperature.

The prepared samples were labeled according to the following nomenclature. The first letter indicates the silica source (H or C), while the second letter denotes the pretreatment applied (W: washed, T: acid treatment). The suffix specifies the amine used for functionalization (TEPA, PEI or PEHA). For example, HW-TEPA corresponds to H silica waste washed and functionalized with TEPA.

### 2.3 Materials Characterization

Geothermal silica waste was characterized by X-ray fluorescence (XRF) and X-ray diffraction (XRD) to determine its chemical and structural composition, respectively. XRF was performed on a sequential X-ray spectrometer (Rigaku Primus II) equipped with a rhodium tube and a 30 μm beryllium window, with a detection limit of 1 μg/g. XRD analyses was conducted using a Bruker AXS D8 Advance powder diffractometer with a Cu Kα radiation source (λ = 1.5406 Å). Additionally, specific surface area (BET model) and pore size distribution (BJH model) of silica samples, before and after citric acid treatment, were determined by N_2_ physisorption using a Belsorp II mini equipment at −196 °C. Before analysis, silica samples were cleaned by degassing overnight at 200 °C under N_2_ flow.

After the amine impregnation process, spectroscopic analysis (FT-IR) was performed to confirm the presence of amino groups, and the amount of loaded amine was determined by N_2_ decomposition through thermogravimetric analysis. FT-IR spectra were obtained with a Thermo Scientific Nicolet 6700 FT-IR spectrometer equipped with an attenuated total reflection (ATR) module. A Labsys Evo Setaram thermobalance was used for N_2_ decomposition. Twenty mg of each SiO_2_-amine material was placed in the thermobalance. The analyses were carried out over a temperature range of 25 to 400 °C, at a heating rate of 5 °C/min, under a nitrogen atmosphere (60 mL/min).

### 2.4 CO_2_ Capture Test

The adsorption–desorption capacities were determined by thermogravimetric analysis. Before the CO_2_ adsorption test, the samples were pretreated by heating to 100 °C under N_2_ flow for 1 h, and then cooled to the adsorption temperature. CO_2_ adsorption was measured at 25, 30, and 35 °C in a 400 ppm of CO_2_ atmosphere, with N_2_ as the gas balance (PRAXAIR-certified mixture) for 4 h. Finally, the regeneration capacity and stability of the material with the best CO_2_ capture results were evaluated by performing 10 CO_2_ adsorption–desorption cycles.

## 3 Results and Discussion

### 3.1 Materials Characterization

The chemical composition of silica waste, determined by XRF, is presented in Table 2. As expected, SiO_2_ is the main component in both samples, with 94.85 and 83.71 wt % in the H and C samples, respectively. These results also showed that, in the C silica sample, water washing removed impurities by dissolving soluble sodium and potassium salts. Thus, after washing, the SiO_2_ increased to 95.16 and 92.08 wt % in H and C, respectively. Therefore, from this point on, it was decided to continue working with the washed samples.

**Table 2:** Chemical Composition of Geothermal Silica Waste by XRF: Majority Elements

	H silica waste		C silica waste	
component	unwashed (wt %)	washed (wt %)	unwashed (wt %)	washed (wt %)
SiO_2_	94.85	95.16	83.711	92.088
TiO_2_	0.04	0.06	0.019	0.047
Al_2_O_3_	0.26	0.32	0.000	<0.01
Fe_2_O_3_	0.06	0.08	0.065	0.069
MnO	<0.01	<0.01	0.023	0.024
MgO	0.06	0.08	0.055	0.055
CaO	0.15	0.17	0.654	0.529
Na_2_O	0.26	0.27	4.28	0.98
K_2_O	0.14	0.15	0.88	0.208
P_2_O5	0.02	0.02	<0.01	<0.01
LOI^a^	4.14	3.67	10.31	6.000

a LOI: loss on ignition.

The structural characterization of the washed and acid-treated silica waste was performed by XRD. The obtained diffractograms (Figure 1) exhibit typical features of an amorphous phase, as evidenced by a broad, diffuse peak at 2θ ≈ 22°, indicative of a disordered structure.^38^ Additionally, the crystalline phases cristobalite (ICSD 00-001-0424) and tridymite (ICSD 00-003-0227) were identified. The coexistence of these two crystalline SiO_2_ phases with amorphous silica indicates partial crystallization and a heterogeneous structure. Furthermore, after acid treatment, the broad and diffuse peak shape was maintained, with no secondary peaks or changes in the central angle.

**Figure 1:**
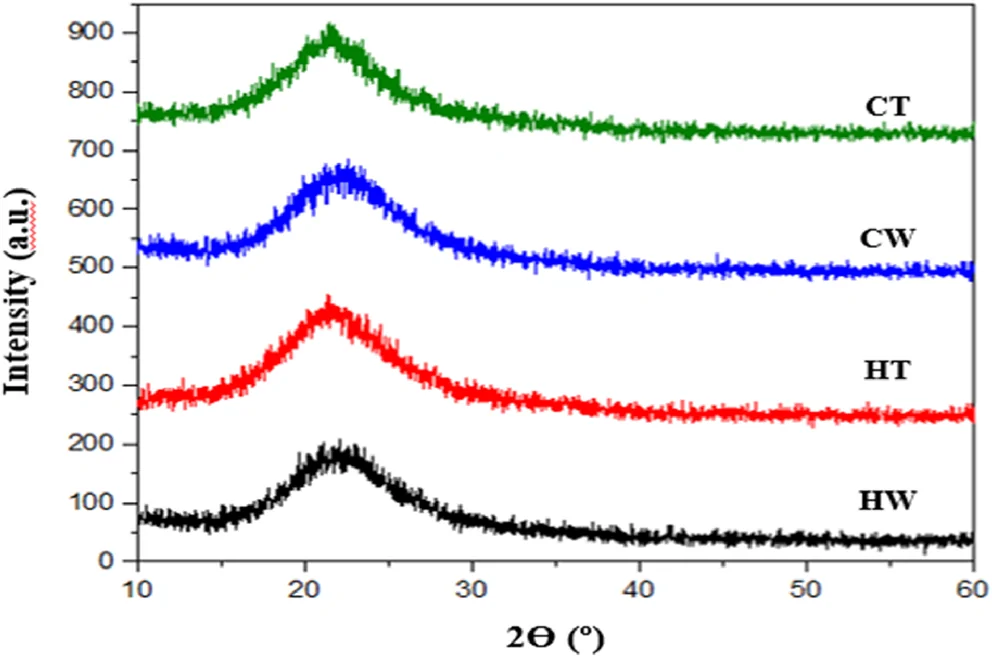
XRD diffractograms of washed and acid-treated silica waste.

The N_2_ adsorption–desorption results are shown in Figure 2. The isotherms of all samples correspond to type II according to the IUPAC classification, which are obtained in nonporous or macroporous solids. H3 hysteresis loops were also observed in the samples studied, as they do not exhibit limited adsorption at high relative pressures and correspond to nonporous solids.^39^ The pore-size distribution described by the BJH model shows that the H silica samples have a predominance of micropores (with an average pore radius of 1.64 nm) and a long tail toward larger pore radius, indicating a heterogeneous distribution. On the other hand, as in the H samples, the pore-size distribution in the C samples is heterogeneous, with predominant radius of 5.27, 14.31, and 22.46 nm for the washed sample (CW), and 10.76, 14.31, and 19.24 nm for the acid-treated (CT); in both cases, the average pore radius is 14.32 nm, which corresponds to a mesoporous structure. It is important to note that the BJH method has limitations when characterizing materials with disordered pore structures. Therefore, the values obtained in this study are interpreted as estimates of the pore-size distribution rather than as a complete description of the pore structure of the materials.

**Figure 2:**
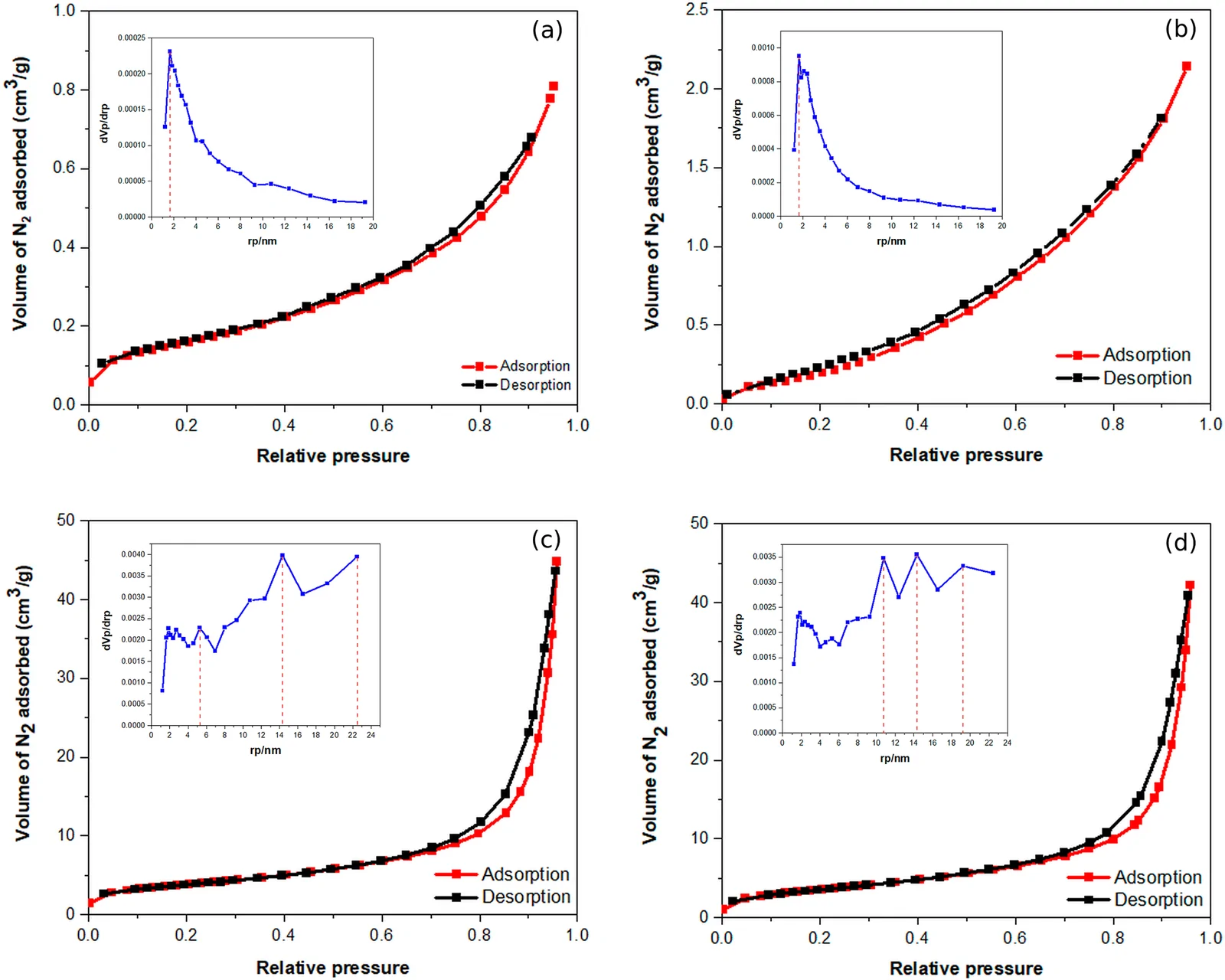
N_2_ adsorption–desorption isotherms and BJH pore-size distribution of silica samples: (a) HW, (b) HT, (c) CW and (d) CT.

Table 3 summarizes the textural properties. In both cases, acid treatment slightly increases the specific surface area and pore volume, suggesting it could improve amine impregnation and CO_2_ adsorption properties. However, these changes are relatively limited, indicating that the acid treatment does not significantly modify the porous structure of the material; instead, its effect is mainly associated with alterations in the surface chemistry of the supports. Incorporating an organic acid during pretreatment introduces oxygen-containing functional groups on the material surface, such as carboxyl groups, whose presence may change surface energy and polarity,^37^ leading to a more chemically favorable surface. These modifications can enhance interactions between the support and the amines during impregnation by improving active-phase dispersion and promoting a more homogeneous distribution across the material surface. Consequently, the effect of the acid treatment on impregnation efficiency is better attributed to improved surface interactions rather than to significant textural development.

**Table 3:** Textural Properties of Washed and Acid-Treated Silica Waste

samples	specific surface area (m^2^/g)	pore volume (cm^3^/g)
HW	0.59	0.0012
HT	1.54	0.0033
CW	12.67	0.0636
CT	13.01	0.0672

After preparing the supports, they were functionalized with 20 wt % TEPA, PEI, or PEHA, and the presence of amine groups was confirmed by FT-IR analysis (Figure 3).

**Figure 3:**
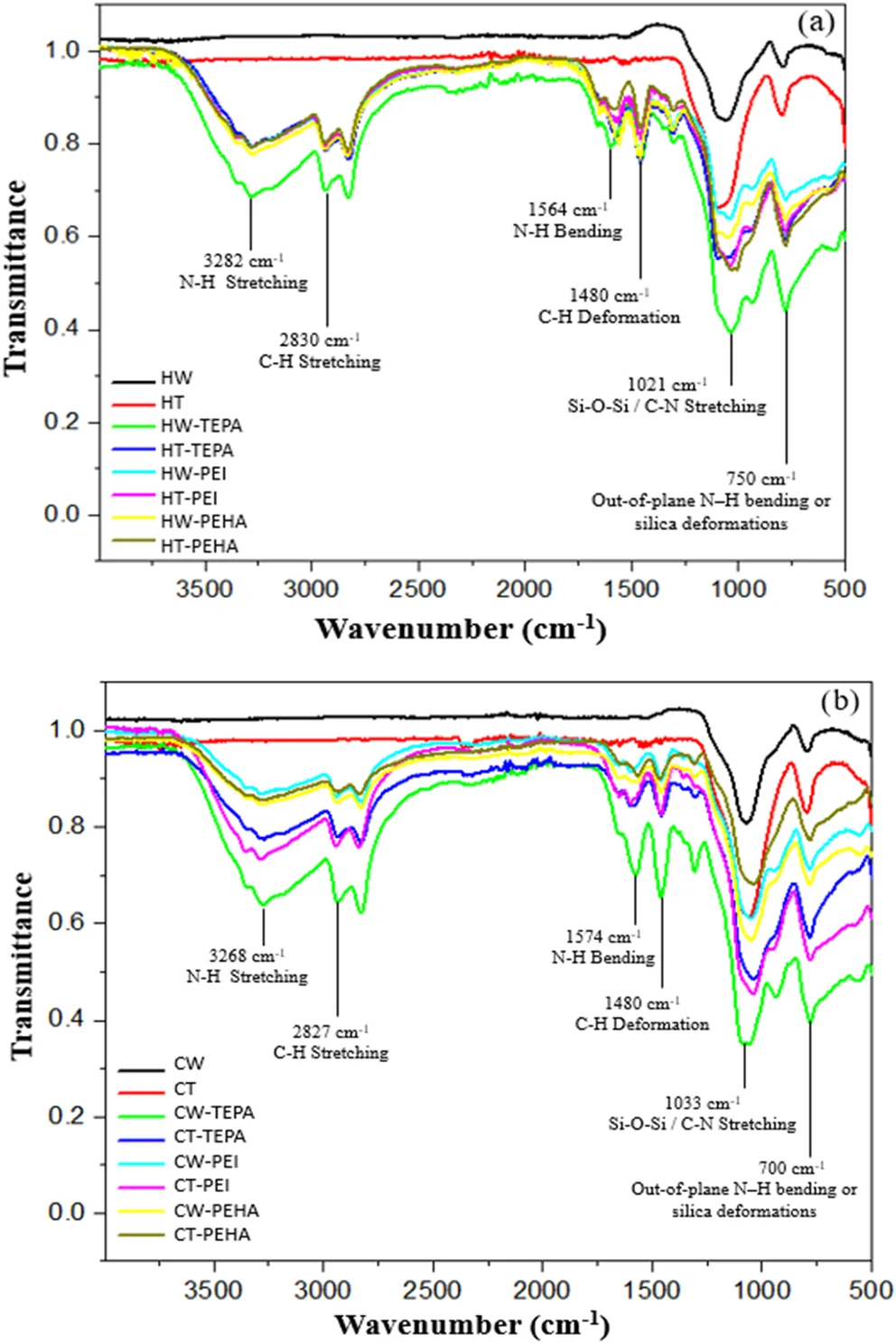
FT-IR spectra of silica-amine materials: (a) H and (b) C.

FT-IR spectra of each silica support were similar, as they result from geothermal processes and show an intense band between 1000 and 1200 cm^–1^, corresponding to Si–O–Si bonds. In the amine-functionalized materials, characteristic stretching vibrations of the N–H group are observed at 3282 and 3268 cm^–1^ in the H and C samples, respectively.^40^ Furthermore, additional bands were observed in all samples that confirm the presence of amines: N–H bending vibrations (1500–1600 cm^–1^) and C–H deformation (1250–1400 cm^–1^).^41^ The signal observed between 2750–2900 cm^–1^ is assigned to C–H stretching, which, although not exclusive to amino groups, supports their presence in the samples.^40^ On the other hand, the band at 1000–1200 cm^–1^ is mainly due to Si–O–Si stretching of the support, although it may overlap with the C–N stretching of the amine, and the 600–800 cm^–1^ band is related to out-of-plane N–H bending vibrations. As shown, these signals appeared in all amine-functionalized materials, but not in the spectra of the supports (black and red lines). Therefore, FT-IR results confirm that the impregnation process was effective.

N_2_ decomposition by thermogravimetric analysis was used to quantify the amount of amine incorporated into the different prepared supports (Figure 4).

**Figure 4:**
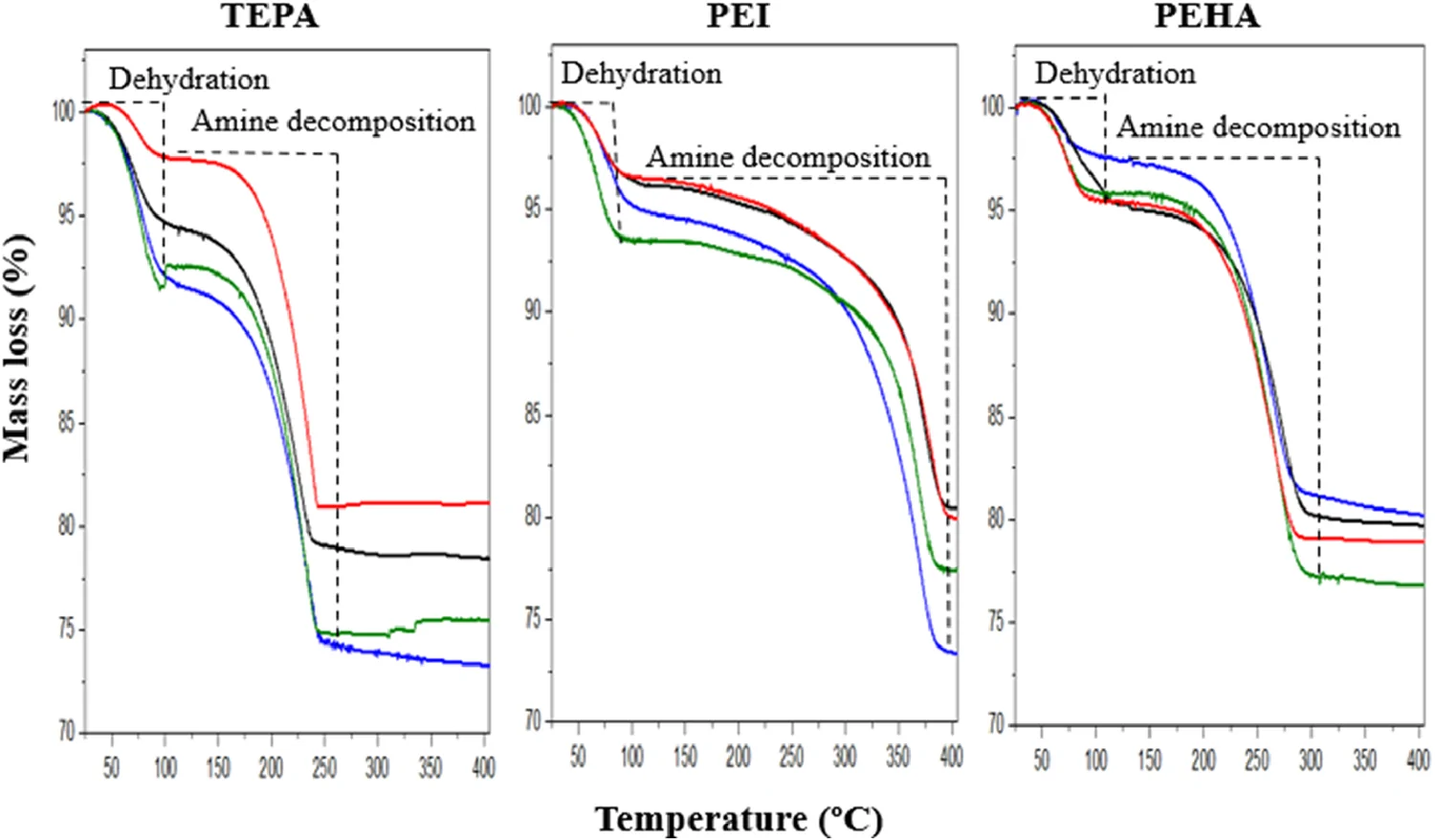
N_2_ decomposition curves of the amine-functionalized materials: HW (black), HT (red), CW (blue), CT (green).

Two main stages of mass loss can be identified: dehydration and amine decomposition. The first stage occurs at low temperatures (30–150 °C) and involves moisture loss, while the second stage involves organic decomposition at higher temperatures. For the amines used in this study, the following decomposition temperature ranges were obtained: TEPA, 150–250 °C; PEI, 200–400 °C; and PEHA, 150–300 °C. Based on Figure 4, the amine load and impregnation efficiency were estimated, as shown in Table 4. Amine loading on all supports was less than 20 wt %, mainly because of their low porosity and specific surface area (Table 3). The amines were more efficiently incorporated into C supports, as their textural properties (specific area and pore volume) are greater than those of the H support. Impregnation efficiencies ranged from 73.57 to 92.80%, which, although not as high as those reported in previous studies, can be considered acceptable, given that the supports used in the present research are waste-derived and had significantly different textural characteristics. As can be observed, the CW-PEI sample exhibited an anomalous impregnation efficiency value (103.93%). This value is physically unrealistic and likely reflects limitations of the estimation method, which is based on mass loss during thermogravimetric analysis. Moisture and the possible removal of weakly retained surface species, such as synthesis residues or adsorbed compounds, may overestimate mass loss.

**Table 4:** Amine Load and Impregnation Efficiency of SiO_2_-Amine Materials

	TEPA	PEI	PEHA
silica support	mass loss/impregnation efficiency (%)	mass loss/impregnation efficiency (%)	mass loss/impregnation efficiency (%)
HW	14.78	15.53	14.71
	73.88	77.63	73.57
HT	16.61	16.32	16.29
	83.03	81.58	81.43
CW	17.05	20.79	15.32
	85.26	103.93	76.62
CT	17.63	15.80	18.56
	88.16	79.00	92.80

### 3.2 Assessment of the CO_2_ Capture Process

To evaluate the CO_2_ capture capacity of the different amine-functionalized materials prepared from geothermal silica waste, isothermal tests were carried out at 25, 30, and 35 °C with a constant CO_2_ concentration of 400 ppm. Figures 5, 6 and 7 show the results expressed as the % mass of CO_2_ adsorbed over time. Additionally, Figure 6 includes an experiment under N_2_ flow (orange line) as a reference to confirm that the mass increase is mainly due to CO_2_ adsorption.

**Figure 5:**
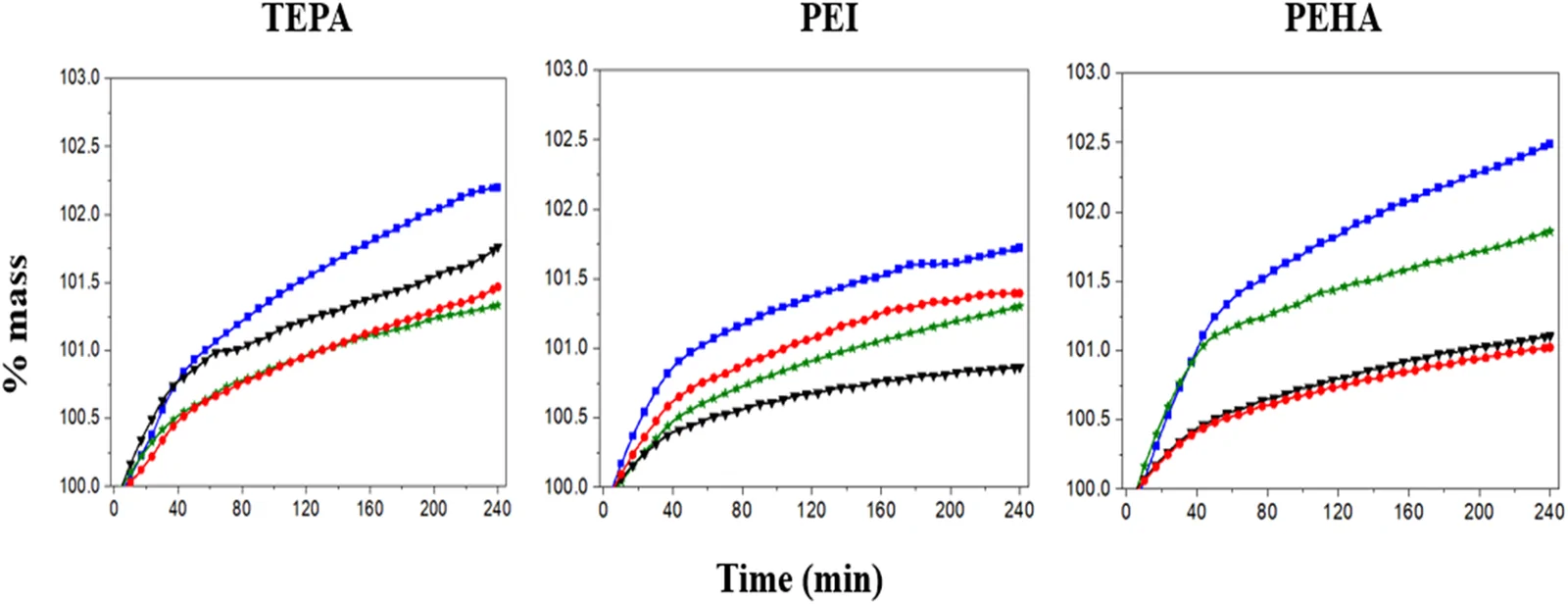
CO_2_ capture of SiO_2_-amine materials at 25 °C and 400 ppm of CO_2_: HW (black), HT (red), CW (blue), CT (green).

**Figure 6:**
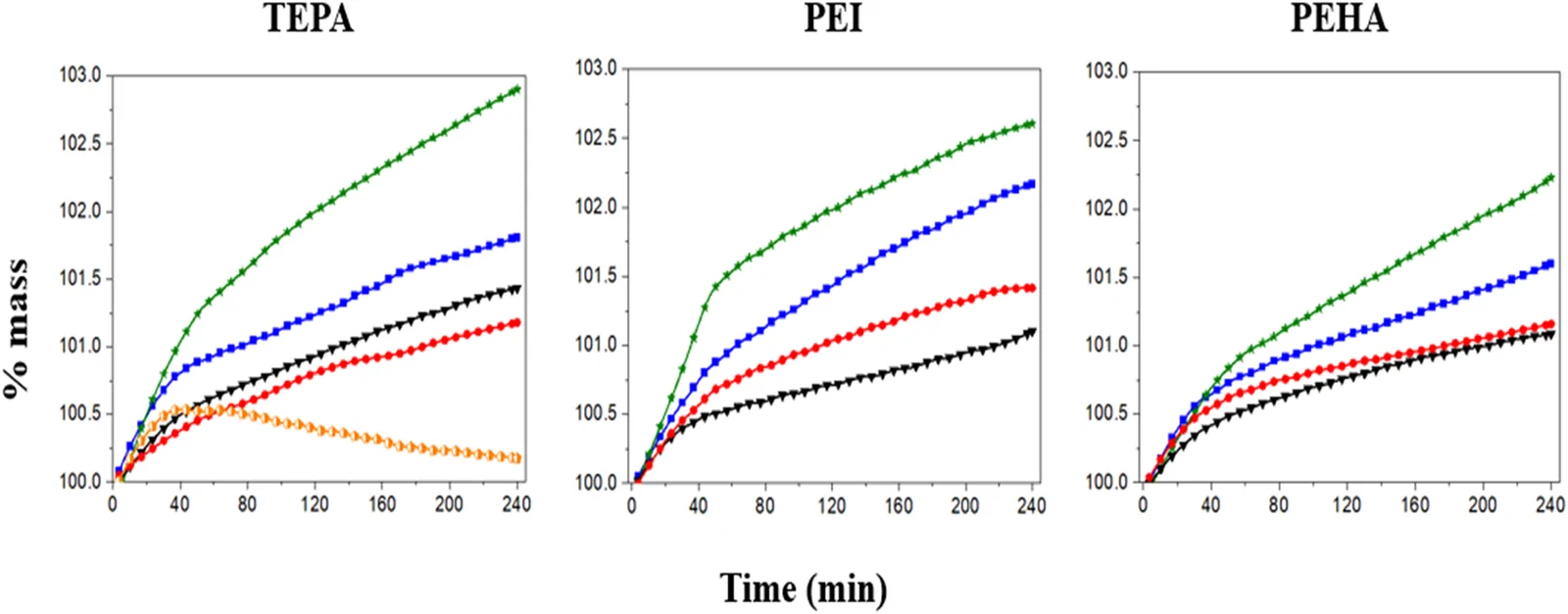
CO_2_ capture of SiO_2_-amine materials at 30 °C and 400 ppm of CO_2:_ HW (black), HT (red), CW (blue), CT (green). CT under N_2_ flow (orange).

**Figure 7:**
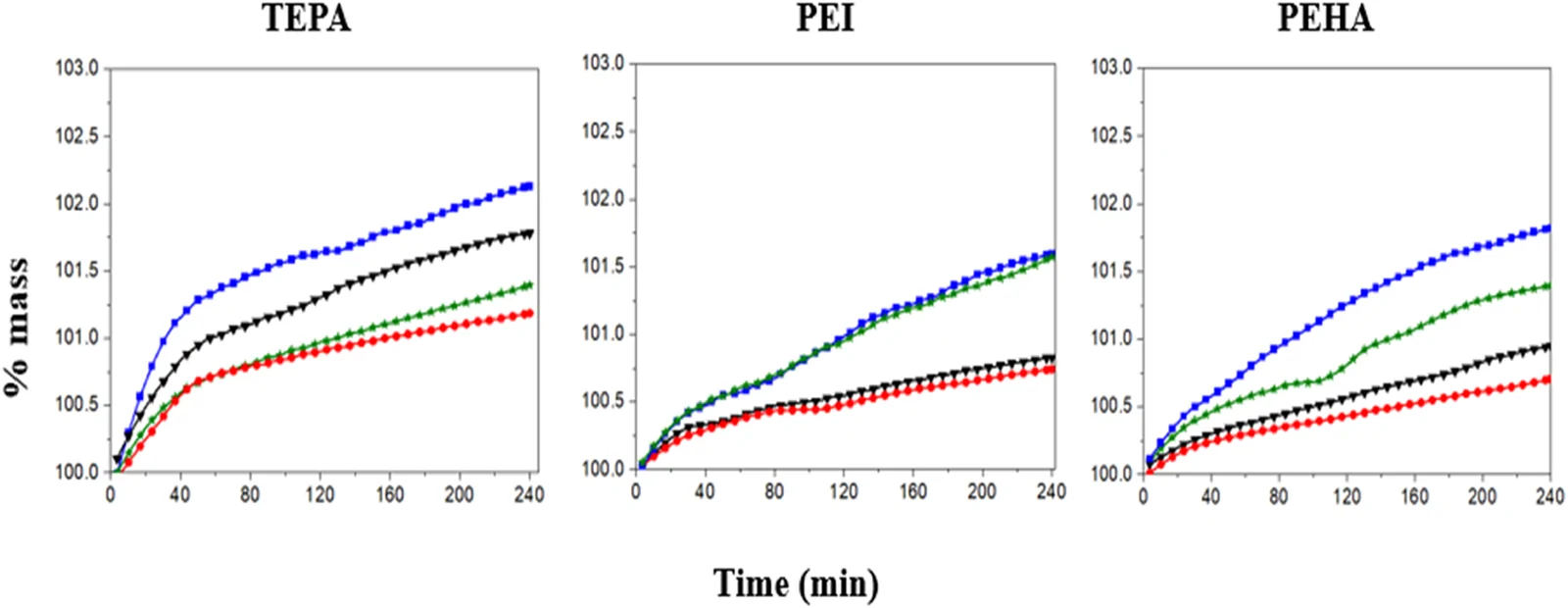
CO_2_ capture of SiO_2_-amine materials at 35 °C and 400 ppm of CO_2_: HW (black), HT (red), CW (blue), CT (green).

The isotherms exhibit exponential behavior, with rapid adsorption at the beginning of each test that slows over time due to adsorbent saturation and slow diffusion between particles. At the start of the experiments, the greater availability of active sites on the external surface of the adsorbent allows CO_2_ to adhere more readily. As these sites become occupied, their availability decreases, reducing the adsorption rate. On the other hand, the limited porosity of the materials makes gas diffusion between adsorbent particles a significant factor in the decrease in adsorption. Diffusion kinetics are also affected by the concentration gradient, which is high at the beginning of the process because the CO_2_ concentration in the gas is high and in the solid is zero, leading to rapid diffusion toward the solid surface. Over time, this gradient will decrease as the material adsorbs CO_2_ and its concentration increases, while the gas concentration decreases. Therefore, the diffusion rate and CO_2_ capture process slow as the system approaches equilibrium. However, equilibrium was not reached during the 4-h experiment; more time is needed to reach equilibrium and determine the maximum CO_2_ capture capacity of each SiO_2_-amine material under the different experimental conditions.

At the experimental conditions evaluated in this work, the highest CO_2_ adsorption was observed at 30 °C, suggesting that gas diffusion over the surface is greater at this temperature. At 25 °C, the CO_2_ capture process becomes less efficient because molecules have lower kinetic energy. In contrast, at 35 °C, CO_2_ capture decreases, likely because CO_2_ desorbs more readily or because the amine efficiency is reduced by increased thermal agitation.^42^

TEPA-impregnated materials achieved the highest CO_2_ uptake compared with PEI- or PEHA-impregnated materials. To quantitatively compare the different amines, amine utilization efficiency was estimated as the molar ratio of adsorbed CO_2_ to the nitrogen content in the adsorbent (mol CO_2_/mol N). The calculated values ranged from 0.040 to 0.161 mol CO_2_/mol N. CT-TEPA showed the highest CO_2_ uptake (0.65 mmol CO_2_/g), corresponding to a nitrogen utilization efficiency of 0.140 mol CO_2_/mol N. In contrast, CT-PEI showed the highest nitrogen utilization efficiency (0.163 mol CO_2_/mol N), although its CO_2_ uptake was slightly lower (0.59 mmol CO_2_/g). These results show that CO_2_ adsorption capacity depends not only on the amine groups theoretically available to react with CO_2_ molecules but also on the combined effect of several factors, among which the molecular structure of the amine plays a key role, as steric hindrance limits diffusion. TEPA is a linear amine with the smallest molecular size among the three studied, allowing better dispersion within the materials, less pore blocking, and greater CO_2_ diffusion.^43^ It is also important to consider the predominant amino group in each structure and how its presence influences the capture process. Adsorbents prepared with TEPA and PEHA (composed only of primary and secondary amines) are more efficient for CO_2_ capture under dry conditions than those impregnated with PEI (which contains all three types of amino groups).

Regarding the origin of the adsorbents, those prepared from C geothermal waste achieved the highest CO_2_ adsorption. Although these materials exhibit a higher specific surface area and pore volume, their superior performance cannot be attributed solely to their textural properties. In addition, C-derived materials showed higher impregnation efficiencies, which resulted in greater CO_2_ capture capacity, as higher amine loadings provide more groups available to interact with CO_2_.

On the other hand, the effect of citric acid treatment on CO_2_ capture cannot be explained solely by the slight increase in surface area observed after treatment. For materials derived from C silica waste, the increase in CO_2_ capture at 30 °C suggests that the treatment mainly modifies the surface chemistry rather than the porous structure. As discussed previously, introducing oxygen-containing functional groups can enhance interactions between the support and the amines, promoting more homogeneous dispersion and greater accessibility of active sites. Consequently, the improved performance of the treated materials is primarily attributed to changes in surface chemistry rather than to significant modifications in textural properties.

Figure 8 summarizes the amount of CO_2_ captured by each adsorbent material during the isothermal tests at different temperatures. At 30 °C, CT-TEPA and CT-PEI reached the highest capture capacities in the data set (0.65 and 0.59 mmol CO_2_/g, respectively), suggesting that at 30 °C, a better balance is achieved among amine distribution, textural properties, and chemical affinity toward CO_2_. For the adsorbents derived from H silica waste, HT-PEI and HT-PEHA, a slight increase in CO_2_ adsorption was observed at 25 °C (0.31 and 0.26 mmol CO_2_/g, respectively). These results indicate that the chemical treatment in H samples does not consistently enhance capture performance. CT-TEPA showed the highest CO_2_ adsorption at 30 °C (0.65 mmol/g), while HT-PEI and HT-PEHA showed the lowest at 35 °C (0.17 mmol/g). These results confirm that adsorbent effectiveness depends on interactions among the support origin, the applied treatment, the type of impregnated amine, and the operating temperature.

**Figure 8:**
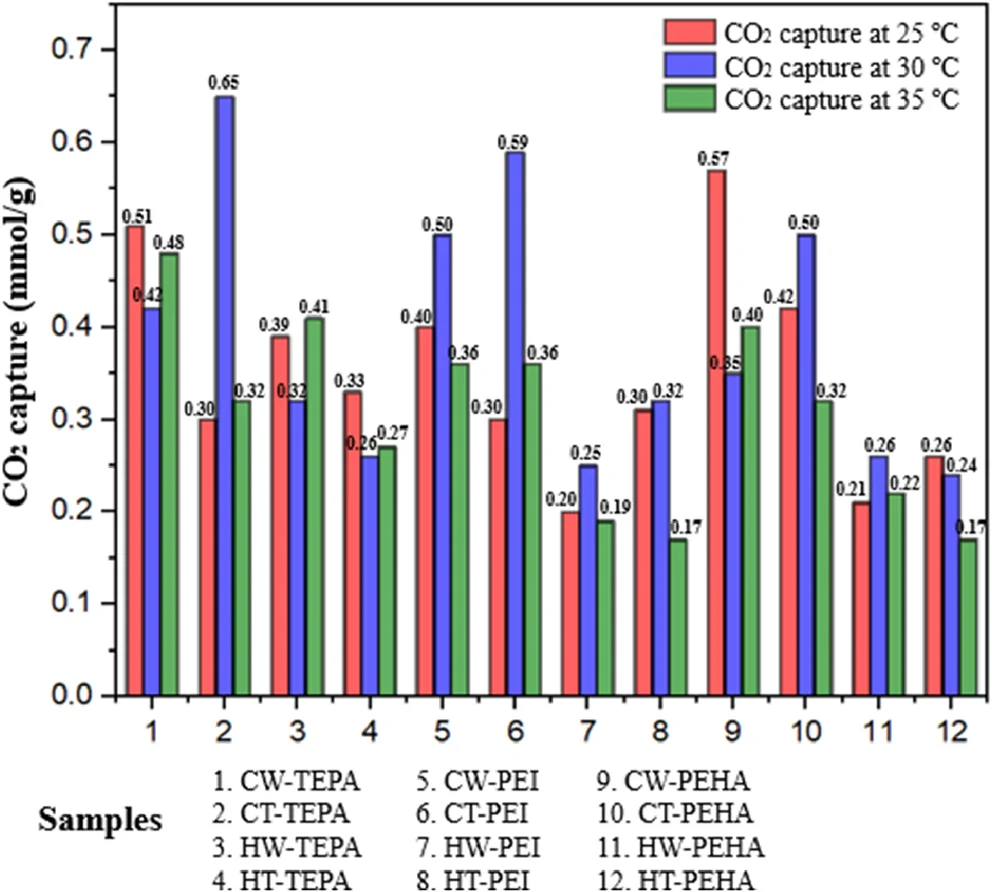
Comparison of the CO_2_ capture of all SiO_2_-amine materials: at 25, 30, and 35 °C and 400 ppm of CO_2_.

To corroborate carbamate formation, a product of chemical adsorption of CO_2_ on amine-modified solid adsorbents, an FT-IR analysis was performed on the material with the best CO_2_ capture performance under the study conditions. The CT-TEPA material after CO_2_ adsorption at 30 °C was selected, and FT-IR spectra before and after CO_2_ capture are shown in Figure 9. A slight increase in the intensity of the 1590 and 1300 cm^–1^ bands can be observed, which correspond to C=O stretching vibrations and C–O bond vibrations, respectively. These bands confirm carbamate formation after CO_2_ capture with amine-modified solid adsorbents.

**Figure 9:**
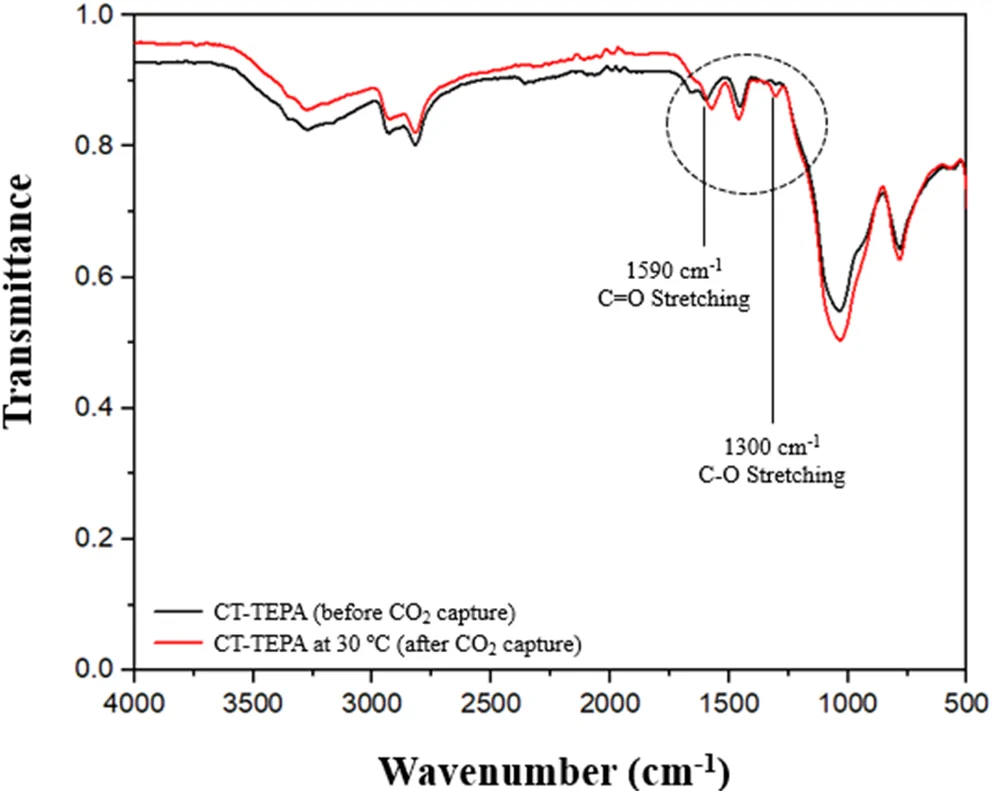
FT-IR spectra of CT-TEPA material before (black) and after CO_2_ capture at 30 °C (red).

To contextualize the results, Table 5 compares them with other waste-derived DAC adsorbents reported in the literature.

**Table 5:** Comparative Summary of CO_2_ Capture of Adsorbents from Industrial Waste in DAC Conditions

waste source	material	adsorption conditions (*T*_ads_ (°C)/ppm of CO_2_)	CO_2_ uptake (mmol CO_2_/g)	refs
bamboo biochar	activated carbon	25 °C/400 ppm	0.052	^29^
sugar cane bagasse	activated carbons	500 ppm	0.70	^30^
corn straw			0.75	
sawdust			0.71	
municipal wastewater sludge	activated carbon – 20 wt % TEPA	environmental conditions	1.17	^31^
geothermal silica	CT – 20 wt % TEPA	30 °C/400 ppm	0.65	this study

As shown, the CT-TEPA material developed in this work exhibits a CO_2_ adsorption capacity of 0.65 mmol/g, which falls within the range reported for adsorbents synthesized from waste materials. The performance of these materials is closely related to the nature and composition of the precursor, which influence the development of the porous structure (surface area, pore volume, and pore size distribution). This is particularly relevant for amine-functionalized systems, where amine dispersion efficiency and the degree of pore blockage are critical factors, as they determine the availability of active sites for CO_2_ interaction under low-concentration conditions. Although waste-derived materials have lower adsorption capacities than adsorbents synthesized from analytical-grade precursors, they offer a sustainable alternative by reducing synthesis costs and the environmental impacts of waste disposal. Furthermore, their versatility creates opportunities to optimize their physicochemical properties, offering significant potential to improve performance in direct air capture applications.

### 3.3 Kinetic Analysis

The kinetic analysis of the experimental data was performed with the pseudo-second-order (PSO) model. Initially, the linearized form was evaluated.^44^

1 $$\frac{t}{q_{t}}=\frac{1}{k_{2}\cdot {q_{e}}^{2}}+\frac{t}{q_{e}}$$

However, the linearized representation had limitations in describing experimental behavior because of mass-transfer issues, particularly during the initial stages of adsorption. Therefore, the kinetic parameters were determined by nonlinear least-squares regression of the experimental data to the PSO model:^45^

2 $$q_{t}=\frac{k_{2}\cdot {q_{e}}^{2}\cdot t}{1+k_{2}\cdot q_{e}\cdot t}$$

Where *q*_*t*_ and *q*_e_ represent the amount of CO_2_ adsorbed at time *t* and at equilibrium, respectively, and *k*_2_ is the kinetic rate constant of the model. The initial adsorption rate was calculated as $h=k_{2}\cdot {q_{e}}^{2}$ , while the fit of the experimental data to the model was quantified using the coefficient of determination *R* ^2^ and the root-mean-square error (RMSE). Table 6 presents the kinetic parameters (and calculated statistical metrics) of C silica-amine materials, which exhibited the highest CO_2_ capture values.

**Table 6:** Kinetic Parameters of CT Silica-Amine Materials Obtained by the PSO Model

material	temperature (°C)	*q*_e_ (mmol/g)	*k*_2_ (g/mmol·min)	*h* (mmol/g·min)	*R* ^2^	RMSE (mmol/g)
CT-TEPA	25	0.3924	0.0168	0.0026	0.9937	0.0050
CT-TEPA	30	0.8626	0.0087	0.0065	0.9853	0.0170
CT-TEPA	35	0.3696	0.0213	0.0029	0.9688	0.0106
CT-PEI	30	0.6086	0.0234	0.0087	0.9805	0.0162
CT-PEHA	30	0.8107	0.0060	0.0039	0.9837	0.0143

The nonlinear PSO model satisfactorily described the experimental curves, with *R* ^2^ values ranging from 0.9688 to 0.9937 and RMSE values ranging from 0.0050 to 0.0170 mmol/g. These results indicate good agreement between the experimental values and the model-calculated values.

Figure 10 compares the experimental curves with the model-calculated values as a function of temperature and amine type, respectively.

**Figure 10:**
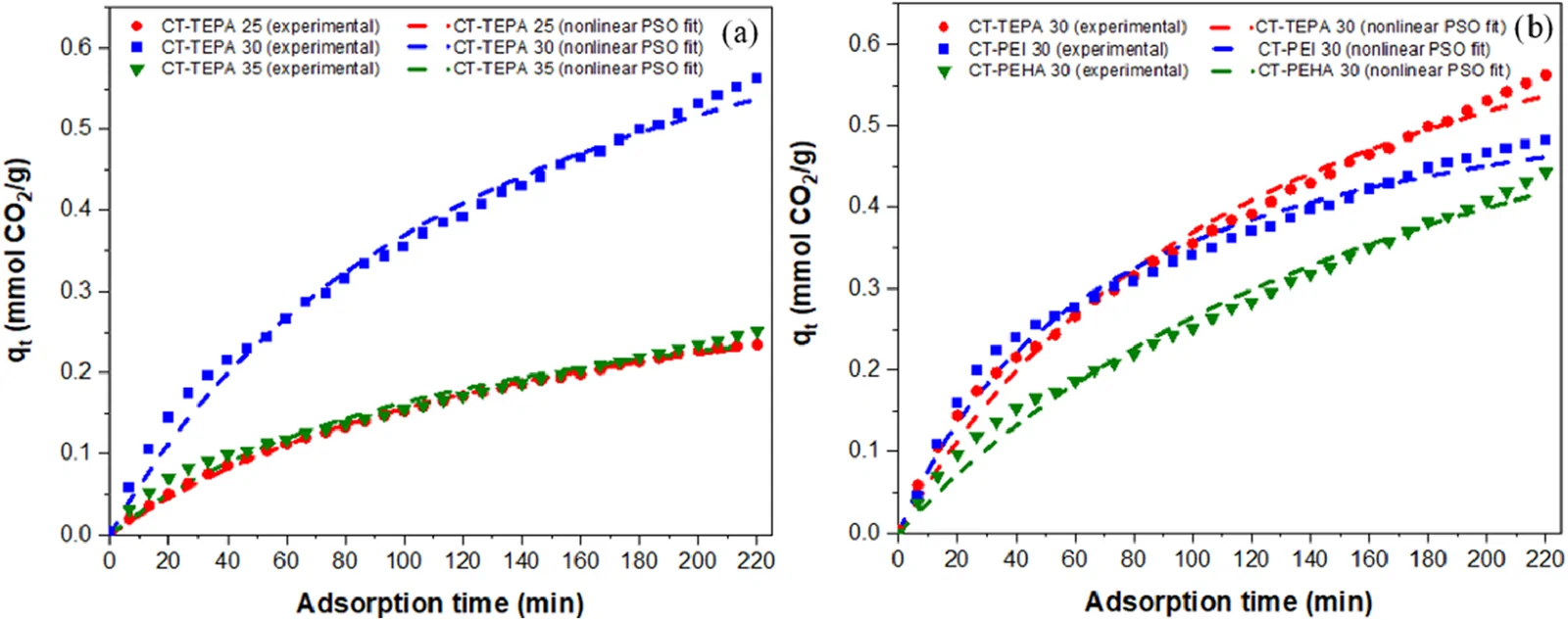
Experimental CO_2_ adsorption curves and corresponding fits to the nonlinear PSO kinetic model: (a) effect of temperature (25, 30, and 35 °C) on CO_2_ capture of CT-TEPA and (b) effect of amine type on CO_2_ capture of CT-amine materials at 30 °C.

For CT-TEPA, *k*_2_ decreased from 0.0168 to 0.0087 g/mmol·min as temperature increased from 25 to 30 °C, while the initial adsorption rate increased from 0.0026 to 0.0065 mmol/g·min. This behavior indicates that increasing the temperature from 25 to 30 °C favored the initial CO_2_ capture rate because the model predicted a higher equilibrium adsorption capacity. In contrast, at 35 °C, although the highest *k*_2_ value (0.0213 g/mmol·min) was obtained, the initial adsorption rate remained low (0.0029 mmol/g·min) because of the lower estimated *q*_e_. Therefore, under the studied conditions, 30 °C provided the most favorable initial kinetic behavior for CO_2_ capture.

Regarding the amine incorporated into the support, CT-PEI at 30 °C showed the highest *k*_2_ value (0.0234 g/mmol·min) and the highest initial adsorption rate (0.0087 mmol/g·min). CT-TEPA showed an intermediate initial adsorption rate (0.0065 mmol/g·min), whereas CT-PEHA presented the lowest value (0.0039 mmol/g·min), indicating slightly slower initial adsorption. These results suggest that the amine type influences CO_2_ capture kinetics, which may reflect differences in active-site accessibility or CO_2_ transport within the amine-containing phase.

Finally, the possible contribution of transport phenomena was evaluated using the Weber–Morris intraparticle diffusion model:^46^

3 $$q_{t}=k_{id}\cdot t^{1/2}+C$$

Where *k*_id_ represents the intraparticle diffusion rate constant, and *C* is a constant related to the thickness of the fluid film (also referred to as the boundary layer) surrounding the adsorbent surface. Theoretically, if the plot of *q*_*t*_ vs *t* ^1/2^ yields a straight line passing through the origin, intraparticle diffusion can be considered the rate-controlling step of the adsorption process. Table 7 presents the kinetic parameters obtained from the model and the corresponding calculated statistical metrics.

**Table 7:** Kinetic Parameters of CT Silica-Amine Materials Obtained by the Intraparticle Diffusion Model

material	temperature (°C)	*k*_id_ (mmol/g·min^1/2^)	*C* (mmol/g)	*R* ^2^
CT-TEPA	25	0.0178	–0.0260	0.9951
CT-TEPA	30	0.0400	–0.0407	0.9951
CT-TEPA	35	0.0171	–0.0120	0.9894
CT-PEI	30	0.0328	0.0094	0.9790
CT-PEHA	30	0.0319	–0.0540	0.9894

For CT-TEPA, *k*_id_ increased from 0.0178 mmol/g·min^1/2^ at 25 °C to 0.0400 mmol/g·min^1/2^ at 30 °C, then decreased to 0.0171 mmol/g·min^1/2^ at 35 °C, which is consistent with the observed increase in the initial adsorption rate for the PSO model at 30 °C. At 30 °C, CT-TEPA presented the highest *k*_id_, followed by CT-PEI (0.0328 mmol/g·min^1/2^) and CT-PEHA (0.0319 mmol/g·min^1/2^). Since *k*_id_ represents the slope of the *q*_*t*_ versus *t* ^1/2^ relationship, these results indicate differences in the apparent contribution of diffusive transport under the evaluated conditions. The higher *k*_id_ for CT-TEPA at 30 °C suggests a greater apparent contribution of diffusive transport at this temperature compared to the other amines.

The Weber–Morris fits yielded *R* ^2^ values ranging from 0.9790 to 0.9951. However, the intercepts were nonzero in all cases, indicating that the fitted lines did not pass through the origin. Consequently, intraparticle diffusion may contribute to the kinetic behavior but cannot be considered the sole rate-controlling process. This suggests involvement of other mass-transfer phenomena, such as external CO_2_ transport and chemical interaction between CO_2_ and the active sites of the material.

The kinetic analysis showed that the nonlinear PSO model adequately described the temporal evolution of CO_2_ capture. In contrast, the Weber–Morris model indicated a contribution from diffusion phenomena but did not identify intraparticle diffusion as the sole rate-controlling process.

### 3.4 Stability of the Selected Adsorbent during Consecutive CO_2_ Adsorption–Desorption Cycles

To evaluate the stability of the adsorbents, CT-TEPA, the material with the best CO_2_ capture behavior under the study conditions, was subjected to ten consecutive CO_2_ adsorption–desorption cycles, in which the CO_2_ capture was carried out for 4 h at 30 °C with a constant CO_2_ concentration of 400 ppm, while the desorption step lasted 30 min at 110 °C in N_2_ flow (Figure 11). The initial CO_2_ capture decreased from 0.57 mmol CO_2_/g in the first cycle to 0.44 mmol CO_2_/g in the tenth cycle, with an approximate adsorbent loss of 3.09% associated with partial amine degradation during cycling. Nevertheless, CO_2_ adsorption over the ten cycles remained relatively stable, indicating good adsorbent regenerability under the established conditions.

**Figure 11:**
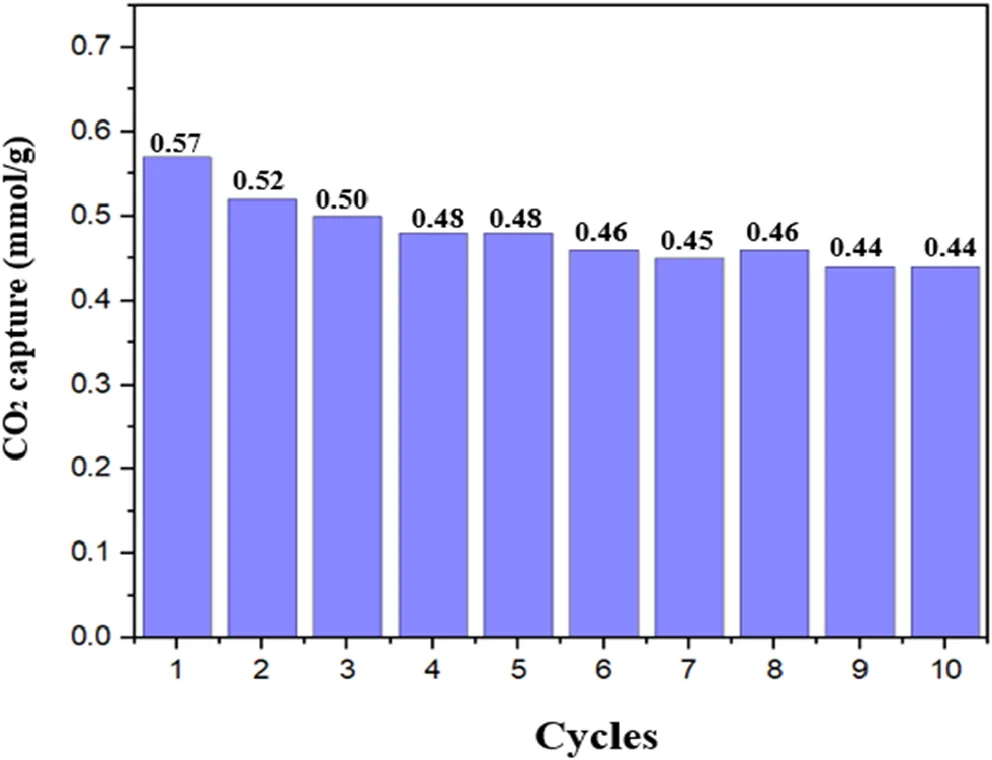
CO_2_ adsorption–desorption behavior of the CT-TEPA material for ten consecutive cycles.

## 4 Conclusions

Both geothermal waste from H and C have a high silica content (>90%) and predominantly amorphous structures. Citric acid treatment increased the specific area and pore volume in both cases. SiO_2_-amine materials were synthesized via wet impregnation, and spectroscopic and thermogravimetric analyses confirmed successful functionalization. Materials prepared from C silica waste show superior impregnation capacity due to their textural and chemical properties after acid treatment.

All SiO_2_-amine materials showed the best CO_2_ capture performance under DAC conditions at 30 °C. CT-TEPA achieved the highest CO_2_ uptake (0.65 mmol/g), and FT-IR analysis confirmed carbamate formation. The nonlinear PSO model adequately described the evolution of CO_2_ adsorption *(R* ^2^ > 0.9680). Kinetic analysis showed that temperature and amine type influenced CO_2_ capture kinetics. For CT-TEPA, the highest initial adsorption rate was observed at 30 °C, whereas increasing the temperature to 35 °C resulted in a lower initial CO_2_ capture rate despite the higher *k*_2_ value. At 30 °C, CT-PEI exhibited the highest initial adsorption rate, followed by CT-TEPA, while CT-PEHA showed the lowest initial adsorption rate. Stability assessment of CT-TEPA over 10 consecutive adsorption–desorption cycles revealed relatively stable performance, indicating good regeneration capacity. However, the material showed a 3.09% mass loss due to amine decomposition. Therefore, further evaluation is needed to understand its behavior over repeated use.

The results demonstrate that geothermal waste is a viable source of silica for developing amine-functionalized adsorbents for DAC conditions. This approach not only advances sustainable direct CO_2_ capture technologies but also enables the valorization of industrial waste in line with circular-economy principles.
